# Epithelioid Sarcoma Mimicking Mycotic Granulomatosis

**DOI:** 10.31662/jmaj.2024-0403

**Published:** 2025-03-07

**Authors:** Yoichi Kaneuchi, Michiyuki Hakozaki, Takuya Nikaido, Yoshihiro Matsumoto

**Affiliations:** 1 Department of Orthopaedic Surgery, Fukushima Medical University School of Medicine, Fukushima, Japan

**Keywords:** epithelioid sarcoma, refractory skin ulcer, groin

A 51-year-old man was referred to our hospital with a 2-year history of a slow-growing refractory skin ulcer on his right groin. The physical examination showed a painless ulcer with firm edges, measuring 6 cm × 2 cm (Figure 1A), but no inguinal lymphadenopathy. The laboratory examination revealed a white cell count of 5,500/mL (reference range, 3,300-8,600/mL) and the C-reactive protein level of 0.12 mg/L (reference range, <0.30 mg/L). Magnetic resonance imaging (MRI) showed an infiltrative superficial soft-tissue mass appearing homogenous and low intensity on T1-weighted images (Figure 1B), heterogeneous with low and iso-intensity on T2-weighted images, and heterogenous on gadolinium-enhanced T1-weighted fat-suppression images. Mycotic granulomatosis was clinically suggested, and a skin biopsy was performed. Bacterial, fungal, and mycobacterium cultures were all negative. The histopathological examination after a second biopsy revealed large, ovoid epithelioid cells with rich eosinophilic cytoplasm (Figure 1C). The immunohistochemical examination revealed that the tumor cells were positive for pan-cytokeratin, CD34, and Friend leukemia integration 1 transcription factor but negative for BAF47/integrase interactor 1 (Figure 1D), leading to the diagnosis of epithelioid sarcoma, which is an extremely rare malignant soft-tissue tumor ^(1)^. The patient underwent a wide resection with a right orchiectomy. Antibiotics were administered long term, but owing to a surgical site infection at one month postoperatively, debridement and placement of antibiotic-loaded cement beads were performed, causing the resolution of the infection. Truncal computed tomography and pelvic MRI were performed every 3 months until 2 years postoperatively, and imaging examinations were continued every 4 months thereafter. At 4 years postoperatively, there is no evidence of metastasis or recurrence. Localized epithelioid sarcoma shows a relatively high overall survival rate of 62%-88%, whereas the rate decreases 24% in patients with metastasis ^(2)^. Lymph node dissemination and metastasis rates for localized epithelioid sarcoma are 34%-52% and 33%, respectively. Epithelioid sarcoma is extremely rare but should be considered as a differential diagnosis when a skin ulcer does not heal over a long period.

**Figure 1.**
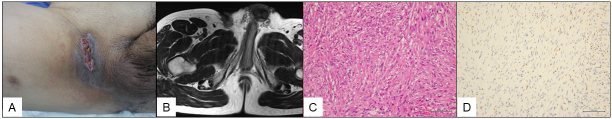
(A) A painless ulcer with firm edges on the right groin, measuring 6 cm × 2 cm. (B) Axial T1-weighted image showed a homogeneous superficial soft-tissue mass infiltrating into the right groin. (C) Large, ovoid epithelioid cells with rich eosinophilic cytoplasm (HE staining). (D) Complete loss of integrase interactor 1 protein expression in the tumor cells. HE: hematoxylin and eosin; T1: type 1.

## Article Information

### Conflicts of Interest

None

## References

[1] Thway K, Jones RL, Noujaim J, et al. Epithelioid sarcoma: diagnostic features and genetics. Adv Anat Pathol. 2016;23(1):41-9. 26645461 10.1097/PAP.0000000000000102

[2] WHO Classification of Tumours Editorial Board. WHO Classification of Tumours, 5th ed, Vol 3. Lyon (France): International Agency for Research on Cancer; 2020. Soft Tissue and Bone Tumours; p. 294-6.

